# Extremely small and incredibly close: Gut microbes as modulators of inflammation and targets for therapeutic intervention

**DOI:** 10.3389/fmicb.2022.958346

**Published:** 2022-08-22

**Authors:** Antonia Piazzesi, Lorenza Putignani

**Affiliations:** ^1^Multimodal Laboratory Medicine Research Area, Unit of Human Microbiome, Bambino Gesù Children’s Hospital, IRCCS, Rome, Italy; ^2^Department of Diagnostic and Laboratory Medicine, Unit of Microbiology and Diagnostic Immunology, Unit of Microbiomics and Multimodal Laboratory Medicine Research Area, Unit of Human Microbiome, Bambino Gesù Children’s Hospital, IRCCS, Rome, Italy

**Keywords:** inflammation, gut microbiota, fecal microbiota transplant, therapy, probiotics, biological mechanisms, inflammatory diseases

## Abstract

Chronic inflammation is a hallmark for a variety of disorders and is at least partially responsible for disease progression and poor patient health. In recent years, the microbiota inhabiting the human gut has been associated with not only intestinal inflammatory diseases but also those that affect the brain, liver, lungs, and joints. Despite a strong correlation between specific microbial signatures and inflammation, whether or not these microbes are disease markers or disease drivers is still a matter of debate. In this review, we discuss what is known about the molecular mechanisms by which the gut microbiota can modulate inflammation, both in the intestine and beyond. We identify the current gaps in our knowledge of biological mechanisms, discuss how these gaps have likely contributed to the uncertain outcome of fecal microbiota transplantation and probiotic clinical trials, and suggest how both mechanistic insight and -omics-based approaches can better inform study design and therapeutic intervention.

## Introduction

Inflammation is a vital biological function evolved to protect the body from external pathogens and harmful or dying cells. Acute inflammation involves identification of the harmful material, emigration of neutrophils and other immune cells from the blood vessels to the offended tissue, and destroying or phagocytosing the culprit. This natural process is largely beneficial, resolves itself in a matter of hours or days, and is a necessary part of healing. However, when the complex machinery involved in mounting these defenses misinterprets these signals the body can respond inappropriately, resulting in a prolonged inflammatory state referred to as chronic inflammation, which is the hallmark of a wide array of disorders collectively and loosely referred to as inflammatory diseases. Uncovering the numerous players responsible for precipitating this inappropriate inflammatory response is an ongoing but necessary scientific challenge, as it is widely believed that combatting chronic inflammation is at the heart of alleviating many of the symptoms associated with these diseases. One such player that is gaining great attention in recent years is the ecosystem of bacteria, viruses, fungi, and other microbes that inhabit the gut, collectively referred to as the gut microbiota (GM).

The gut harbors hundreds of commensal species that perform a wide array of necessary biological functions and whose presence is vital to human survival (Quigley, 2013). Furthermore, these species live in a balanced ecosystem which, when unbalanced, can have negative consequences on human health, a state known as dysbiosis. The body of evidence suggesting a link between dysbiosis of the GM and inflammatory disorders is vast and has been extensively reviewed in many pathological contexts, such as inflammatory bowel disease (IBD) (Kostic et al., 2014; Putignani et al., 2016; Glassner et al., 2020), cancer (Gagnière et al., 2016; Chen et al., 2017; Lucas et al., 2017; Saus et al., 2019; Cheng et al., 2020), rheumatic arthritis (Scher et al., 2016; Konig, 2020; Zaiss et al., 2021), psoriasis (Myers et al., 2019; Olejniczak-Staruch et al., 2021), metabolic diseases (Manco et al., 2010; del Chierico et al., 2014; Nobili et al., 2019; Montanari et al., 2021), food allergies (Vernocchi et al., 2015; Berni Canani et al., 2019; Bunyavanich and Berin, 2019; Shu et al., 2019), and neuropsychiatric disorders (Petra et al., 2015; Koopman and el Aidy, 2017; Peirce and Alviña, 2019; Ristori et al., 2019, 2020; Rutsch et al., 2020). However, many studies investigating the putative role of the GM in inflammatory disease have been limited to identifying dysbiosis in the gut in affected patients. For example, patients with IBD have been found to have reduced diversity of the GM overall (Manichanh et al., 2006; Pascal et al., 2017; Kowalska-Duplaga et al., 2019; lo Presti et al., 2019; Putignani et al., 2021), as well as either expanded or diminished populations of specific bacterial genera/species (Table 1). Patterns of dysbiosis in the gut have emerged for many inflammatory diseases that are also not intestinal in origin, leading to a bacterial signature of chronic inflammation in multiple pathological contexts (Table 2). While these studies are strongly suggestive, whether the bacteria in question actually drive disease progression, or whether they are simply markers for disease-related dysbiosis, is still a matter of debate.

**TABLE 1:** Summary of bacterial genera/species signatures associated with inflammatory diseases, organized as Phylum (Class).

Bacteria	Disease	Signature	References
**Actinomycetota (Actinomycetia)**			
*Bifidobacterium* spp.	IBD	↓	Fyderek et al., 2009; Golińska et al., 2013
	IBS	↓	Liu et al., 2017; Altomare et al., 2021
*Bifidobacterium adolescentis*	IBD(CD)	↓	Joossens et al., 2011
*Bifidobacterium catenulatum*	IBS	↓	Kerckhoffs et al., 2009
Bacillota (Bacilli)			
*Streptococcus* spp.	IBD (CD)	↑	Fyderek et al., 2009
	CRC	↑	Allali et al., 2015
*Enterococcus faecalis*	IBD (CD)	↑	Golińska et al., 2013; Zhou et al., 2016; lo Presti et al., 2019
*Lactobacillus* spp.	IBD (UC)	↑	Fyderek et al., 2009
	IBS	↕	Carroll et al., 2010; Liu et al., 2017
*Lactobacillus ruminis*	ID	↑	Yamashiro et al., 2017
*Gemella* spp.	CRC	↑	Allali et al., 2015
**Bacillota (Clostridia)**			
*Ruminococcus gnavus*	IBD	↑	Png et al., 2010; Joossens et al., 2011; Hall et al., 2017; Henke et al., 2021
*Ruminococcus torques*	IBD	↑	Png et al., 2010
*Faecalibacterium* spp.	CRC	↓	Wu et al., 2013
*Faecalibacterium prausnitzii*	IBD	↓	Sokol et al., 2008, 2009; Joossens et al., 2011; Rossi et al., 2015
	IBS	↓	Liu et al., 2017
*Clostridium coccoides*	IBS	↑	Parkes et al., 2012
	IBD	↓	Sokol et al., 2009
*Clostridium leptum*	IBD	↓	Sokol et al., 2009
*Eubacterium rectale*	IBS	↑	Parkes et al., 2012
*Parvimonas* spp.	CRC	↑	Allali et al., 2015
*Roseburia* spp.	CRC	↓	Wu et al., 2013
*Roseburia intestinalis*	IBD(CD)	↓	Quan et al., 2018
**Bacillota (Erysipelotrichia)**			
*Bulleidia* spp.	CRC	↑	Allali et al., 2015
**Bacillota (Negativicutes)**			
*Dialister invisus*	IBD(CD)	↓	Joossens et al., 2011
**Bacteroidota (Bacteroidia)**			
*Bacteroides* spp.	CRC	↑	Wu et al., 2013
*Bacteroides fragilis*	CRC	↑	Dejea et al., 2018
	ID	↑	Sears et al., 2008
*Bacteroides vulgatus*	IBD (UC)	↑	Fujita et al., 2002; Shiba et al., 2003; Mills et al., 2022
**Campylobacterota (Campylobacteria)**			
*Campylobacter* spp.	CRC	↑	Warren et al., 2013; Wu et al., 2013; Allali et al., 2015
*Campylobacter concisus*	IBD	↑	Zhang et al., 2009; Man et al., 2010; Mahendran et al., 2011; Mukhopadhya et al., 2011; Chung et al., 2016; Underwood et al., 2016; Yde Aagaard et al., 2020
*Campylobacter jejuni*	Enteritis	↑	Sun et al., 2012
**Fusobacteriota (Fusobacteriia)**			
*Fusobacterium* spp.	IBD	↑	Zhou et al., 2016; Putignani et al., 2021
	CRC	↑	Kostic et al., 2013; Warren et al., 2013; Wu et al., 2013; Allali et al., 2015
**Pseudomonadota (Gammaproteobacteria)**			
Adherent-invasive *Escherichia coli*	IBD	↑	Martin et al., 2004; Conte et al., 2006; Arthur et al., 2012; Schippa et al., 2012; Palmela et al., 2018; Shaler et al., 2019; Costa et al., 2020
	CRC	↑	Arthur et al., 2012; Dejea et al., 2018
Diffusely-adherent *Escherichia coli*	IBD	↑	Mirsepasi-Lauridsen et al., 2019; Walczuk et al., 2019
**Pseudomonadota (Betaproteobacteria)**			
*Eikenella*	CRC	↑	Allali et al., 2015
**Verrucomicrobiota (Verrucomicrobiae)**			
*Akkermansia muciniphila*	IBD	↓	Png et al., 2010; Lopetuso et al., 2020
	IBS	↓	Lopetuso et al., 2020

CD, Crohn’s disease; CRC, colorectal cancer; IBD, inflammatory bowel disease; IBS, irritable bowel syndrome; ID, inflammatory diarrhea; UC, ulcerative colitis. Bidirectional arrows indicate different studies with conflicting results.

**TABLE 2:** Gut microbiota (GM) signature for non-intestinal inflammatory diseases.

Phylum (class)	Species	Disease	Signature	References
**The gut-brain axis**				
Actinomycetota (Actinomycetia)	*Bifidobacterium* spp.	ASD	↓	Iglesias-Vázquez et al., 2020
		AD	↓	Vogt et al., 2017
	*Corynebacterium* spp.	ASD	↑	Strati et al., 2017
Actinomycetota (Coriobacteriia)	*Aldercreutzia* spp.	AD	↓	Vogt et al., 2017
	*Collinsella* spp.	ASD	↑	Strati et al., 2017
Bacillota (Bacilli)	*Gemella* spp.	AD	↑	Vogt et al., 2017
	*Lactobacillus* spp.	ASD	↑	Strati et al., 2017
Bacillota (Clostridia)	*Blautia* spp.	AD	↑	Vogt et al., 2017
	*Coprococcus* spp.	ASD	↓	Iglesias-Vázquez et al., 2020
	*Clostridium* spp.	ASD	↑	Iglesias-Vázquez et al., 2020
		AD	↓	Vogt et al., 2017
	*Dorea* spp.	ASD	↑	Strati et al., 2017
	*Faecalibacterium* spp.	ASD	↑	Iglesias-Vázquez et al., 2020
	*Oscillospira* spp.	PANS	↑	Quagliariello et al., 2018
Bacillota (Erysipelotrichales)	*Turicibacter* spp.	AD	↓	Vogt et al., 2017
Bacillota (Negativicutes)	*Dialister* spp.	ASD	↓	Strati et al., 2017
		AD	↓	Vogt et al., 2017
	*Phascolarctobacterium*	ASD	↑	Iglesias-Vázquez et al., 2020
		AD	↑	Vogt et al., 2017
	*Veillonella* spp.	ASD	↓	Strati et al., 2017
Bacteroidota (Bacteroidia)	*Alistipes* spp.	ASD	↓	Strati et al., 2017
		AD	↑	Vogt et al., 2017
	*Bacteroides* spp.	ASD	↑	Iglesias-Vázquez et al., 2020
		AD	↑	Vogt et al., 2017
		PANS	↑	Quagliariello et al., 2018
	*Odoribacter* spp.	PANS	↑	Quagliariello et al., 2018
	*Parabacteroides* spp.	ASD	↕	Strati et al., 2017; Iglesias-Vázquez et al., 2020
Campylobacterota (Campylobacteria)	*Campylobacter jejuni*	GBS	↑	Louwen et al., 2008; Ramos et al., 2021
Thermodesulfobacteriota (Desulfovibrionia)	*Bilophila* spp.	ASD	↓	Strati et al., 2017
**The gut-liver axis**				
Actinomycetota (Actinomycetia)	*Bifidobacterium* spp.	HCC	↓	Ponziani et al., 2019
		NAFL	↓	Nobili et al., 2018
Bacillota (Bacilli)	*Lactobacillus* spp.	NAFL	↑	Nobili et al., 2018
	*Streptococcus* spp.	Cirrhosis	↑	Ponziani et al., 2019
Bacillota (Clostridia)	*Blautia* spp.	NASH	↑	del Chierico et al., 2014
	*Dorea* spp.	NASH	↑	del Chierico et al., 2014
	*Oscillospira* spp.	NAFL	↓	del Chierico et al., 2014
	*Ruminococcus* spp.	NASH	↑	del Chierico et al., 2014
	*Ruminococcus gnavus*	NAFL	↑	Behary et al., 2021
	*Clostridium bolteae*	NAFL	↑	Behary et al., 2021
	*Veillonella parvula*	NAFL + HCC	↑	Behary et al., 2021
Bacteroidota (Bacteroidia)	*Bacteroides caecimuris*	NAFL + HCC	↑	Behary et al., 2021
	*Bacteroides xylanisolvens*	NAFL	↑	Behary et al., 2021
Verrucomicrobiota (Verrucomicrobiae)	*Akkermansia* spp.	Cirrhosis	↓	Ponziani et al., 2019
		HCC	↑	Ponziani et al., 2019
**The gut-lung axis**				
Actinomycetota (Coriobacteriia)	*Eggerthella lenta*	CF	↓	Vernocchi et al., 2018
Bacillota (Bacilli)	*Streptococcus* spp.	CMA	↑	Mennini et al., 2021
Bacillota (Clostridia)	*Clostridium* spp.	CF	↓	Vernocchi et al., 2018
	*Dorea formicigenerans*	CF	↓	Vernocchi et al., 2018
	*Faecalibacterium prausnitzii*	CF	↓	Vernocchi et al., 2017
		Asthma	↓	Demirci et al., 2019
Bacteroidota (Bacteroidia)	*Bacteroides* spp.	NSCLC	↓	Vernocchi et al., 2020
	*Prevotella* spp.	CMA	↑	Mennini et al., 2021
Pseudomonadota (Gammaproteobacteria)	*Actinobacillus* spp.	CMA	↑	Mennini et al., 2021
	*Haemophilus* spp.	CMA	↑	Mennini et al., 2021
	*Klebsiella* spp.	CMA	↑	Mennini et al., 2021
Verrucomicrobiota (Verrucomicrobiae)	*Akkermansia muciniphila*	NSCLC	↓	Vernocchi et al., 2020
		Asthma	↓	Demirci et al., 2019
**Gut-pancreas-metabolism**				
Actinomycetota (Actinomycetia)	*Actinomyces* spp.	Obesity	↑	del Chierico et al., 2018
Actinomycetota (Coriobacteriia)	*Collinsella aerofaciens*	T2D	↑	Kulkarni et al., 2021
Bacillota (Bacilli)	*Lactobacillus ruminis*	T2D	↑	Kulkarni et al., 2021
Bacillota (Clostridia)	*Faecalibacterium prausnitzii*	Obesity	↑	del Chierico et al., 2018
		T2D	↓	Kulkarni et al., 2021
	*Oscillospira* spp.	Obesity	↓	del Chierico et al., 2018
	*Ruminococcus gnavus*	T2D	↑	Kulkarni et al., 2021
Bacteroidota (Bacteroidia)	*Bacteroides caccae*	Obesity	↓	del Chierico et al., 2018
		T2D	↑	Kulkarni et al., 2021
	*Bacteroides ovatus*	IR	↑	del Chierico et al., 2021
	*Butyricimonas* spp.	T2D	↑	Kulkarni et al., 2021
	*Parabacteroides* spp.	Obesity	↓	del Chierico et al., 2018
**The gut-joint axis**				
Actinomycetota (Actinomycetia)	*Bifidobacterium catenulatum*	Gout	↓	Guo et al., 2016
Bacillota (Clostridia)	*Faecalibacterium prausnitzii*	Gout	↓	Guo et al., 2016
Bacteroidota (Bacteroidia)	*Bacteroides caccae*	Gout	↑	Guo et al., 2016
	*Bacteroides xylanisolvens*	Gout	↑	Guo et al., 2016
	*Prevotella* spp.	RA	↑	Maeda et al., 2016; Kishikawa et al., 2020

AD, Alzheimer’s disease; ASD, autism spectrum disorder; CF, cystic fibrosis; CMA, cow’s milk allergy; GBS, Guillain–Barrè syndrome; HCC, hepatocellular carcinoma; IR, insulin resistance; NAFL, non-alcoholic fatty liver disease; NASH, non-alcoholic steatohepatitis; NSCLC, non-small cell lung cancer; PANS, pediatric acute-onset neuropsychiatric syndrome; RA, rheumatic arthritis; T2D, type-2 diabetes. Bidirectional arrows indicate different studies with conflicting results.

Some studies have attempted to demonstrate a degree of causality between dysbiosis and disease in human studies by different statistical means, such as Mendelian randomization analyses, and other computational models (Sanna et al., 2019; Lv et al., 2021). Others have attempted to address these concerns by employing both preclinical and germ-free murine models. In mice, transplantation of the GM of a preclinical model of colorectal cancer into germ-free mice resulted in intestinal inflammation and tumorigenesis, which was reversed upon treatment with antibiotics (Zackular et al., 2013). Another study found that monocolonization of a germ-free, colitis-susceptible mouse model with *Escherichia coli* NC101 was sufficient to induce intestinal inflammation and tumorigenesis (Arthur et al., 2012). In a study using bacteria from humans, a tumor-prone mouse model co-infected with *Escherichia coli* and *Bacteroides fragilis* strains isolated from patients with familial adenomatous polyposis had a stronger pro-inflammatory response, greater tumor growth, and higher mortality rates than mice infected with either strain alone (Dejea et al., 2018). Beyond the gut, fecal microbiota transplantation between mouse models and the modulation of the GM with either pro- or antibiotics was sufficient to shape the hepatic inflammatory environment and either promote or suppress liver carcinogenesis (Schneider et al., 2022). In humans, similar bacterial species were found to characterize patients who responded positively to hepatic cancer treatment (Ponziani et al., 2022). In these studies, the causal link between gut dysbiosis and inflammatory diseases has at least been strengthened by these methods.

However, there have also been times when studies designed to uncover a causal link between dysbiosis and pathology have not supported the hypothesis that these specific bacteria are drivers of disease progression. It has been demonstrated that not taking confounding variables, such as diet, weight, alcohol consumption, and tobacco use into consideration when matching patients with healthy controls can lead to exaggerated and artifactual findings of dysbiosis (Vujkovic-Cvijin et al., 2020). Consistently, one large metagenomics study performed in Australia concluded that the variability in the GM between patients with autism and healthy controls was due to other confounding variables, such as age and diet, rather than being directly related to autism spectrum disorder (Yap et al., 2021). Furthermore, despite the numerous studies associating the prevalence of *Campylobacter* spp. with inflammatory bowel disease in both adults and children (Table 1) and a meta-analysis predicting the contrary (Castaño-Rodríguez et al., 2017), a longitudinal case study of a Danish cohort found that patients with *Campylobacter concisus*- or *Campylobacter jejuni*-positive stool samples were not at increased risk of developing IBD (Nielsen et al., 2019), nor was there an increased prevalence of *Campylobacter concisus* in a study of British children presenting with *de novo* IBD (Hansen et al., 2013). Despite these disappointing results, it is important to note that these studies do not completely exclude the possibility of a causal link between *Campylobacter* spp. and IBD. Even if the presence of these bacteria in the gut does not increase the risk of disease onset, it is still possible that they are responsible for sustaining the chronic inflammation that is initiated by other means, and are therefore still active participants in the pathology. These kinds of studies demonstrate why a better understanding of biological mechanisms can better inform our interpretation of clinical data.

Taken together, these studies have led to a general consensus that the GM can in fact have a direct effect on human health, and at least participate in the progression of various inflammatory diseases. However, while some of these studies have strengthened the evidence of a causal link between the GM and disease progression, much mechanistic insight into how the GM directly affects inflammation is still lacking. In this review, we focus specifically on what is known about the biological mechanisms employed by microbes in the gut to influence inflammation, identify the current gaps in our knowledge, and discuss how a deeper understanding of mechanistic insight can shape future clinical study design and therapeutic strategies.

## Bacterial invasion of intestinal epithelial cells can drive inflammation

One of the most direct ways that intestinal bacteria can precipitate a pro-inflammatory response is by invading the cells in their environment. *In vitro* models of the intestinal epithelial barrier have shown that many bacteria, such as diffusely adherent *Escherichia coli, Shigella dysenteriae, Fusobacterium varium, Bacteroides vulgatus*, and *Clostridium clostridioforme*, adhere to and are internalized by intestinal epithelial cells, whereupon they stimulate the secretion of tumor necrosis factor-α (TNF-α) (Ohkusa et al., 2009; Gopal et al., 2017; Walczuk et al., 2019). One study found that *Campylobacter concisus*, as well as other *Campylobacter* species associated with Crohn’s disease, were also able to invade human intestinal epithelial cells *in vitro*, avoid being phagocytized, and induce inflammation (Man et al., 2010). Most intriguingly, this invasion was significantly increased upon co-treatment with either TNF-α or interferon-γ (IFN-γ), suggesting that *Campylobacter* species are more virulent in an environment that is already inflamed (Man et al., 2010). In light of the Danish study finding no significant increase in the risk of developing IBD in *Campylobacter concisus*-positive patients (Nielsen et al., 2019), these studies taken together support the hypothesis that *C. concisus* influences IBD progression by sustaining a pro-inflammatory response initiated by other factors. Another (not necessarily exclusive) explanation is the high variability in virulence and intracellular survival between different *C. concisus* strains, with those isolated from patients with chronic IBD having an invasive potential of up to 500 times those isolated from healthy controls (Kaakoush et al., 2011; Deshpande et al., 2013), as well as an increased ability to evade the autophagic process (Burgos-Portugal et al., 2014). Furthermore, the most invasive Campylobacter concisus strains isolated from patients were also found to express exotoxin 9, a gene with a domain that has homologues in many viruses and is thought to contribute to their virulence (Kaakoush et al., 2011). Another possibility for differences in virulence is the expression of zonula occludens toxin (zot), which is only found in a subset of *C. concisus* strains and which alone can induce a pro-inflammatory response, cause epithelial barrier damage, and initiate cell death *in vitro* (Mahendran et al., 2016). Genetic manipulation of different *C. concisus* strains could shed further light on which genes most affect their ability to invade, avoid autophagy, and induce inflammation in human cells. Finally, it is important to note that characterization of the GM rarely resolves down to the species level, let alone strain. Longitudinal studies, such as the ones conducted on Danish adults and British children, which fail to take differences between strains into consideration, may have missed an association between *C. concisus* infection and IBD due to this inherent variability. With recent advances in -omics methods, human studies would ideally include a deeper characterization of the GM to avoid masking such effects.

Similarly, *Campylobacter jejuni* is also associated with intestinal inflammation, enteritis, and colorectal cancer and has long been known to be able to invade the colonic mucosa in humans (van Spreeuwel et al., 1985). Infection of a colitis-susceptible mouse model with *C. jejuni* activated mammalian target of rapamycin (mTOR) signaling, intestinal inflammation, neutrophil infiltration, and severe colitis, which could be reversed by administering rapamycin and activating autophagy (Lippert et al., 2009; Sun et al., 2012). *C. jejuni* also expresses surface lipooligosaccharides (LOS), which mimick human gangliosides, the structure of which varies between strains and are important to the bacteria’s invasive potential and survival both *in vitro* and *in vivo* (Guerry et al., 2002; Louwen et al., 2008; Naito et al., 2010). Genetic manipulation of *C. jejuni* demonstrated that their ability to invade host cells was also strongly correlated with the production of chemotaxis protein CheY and, to a lesser extent, the energy taxis protein CetA (Bouwman et al., 2014). Some strains also produce the genotoxin cytolethal distending toxin, which was shown to be necessary for *C. jejuni*-induced tumorigenesis in germ-free mice, though its absence did not impair its ability to invade host cells (Bouwman et al., 2014; He et al., 2019). Given the many different molecular players involved in the virulence, pro-inflammatory potential and host response to *C. jejuni* infection, deeper strain-level characterization of the GM in human patients could shed more light on the association between *C. jejuni* and inflammatory diseases.

## Bacterial cell-surface molecules can independently influence inflammation

Bacteria can express a variety of molecules on their cell surface, which can aid in motility, intercellular communication, and otherwise influence their microenvironment. In the context of intestinal inflammation, many of these extracellular molecules can also aid in the bacterium’s ability to invade host cells. Cytolethal distending toxin produced by *C. jejuni*, for example, is composed of three subunits, two of which bind lipid rafts on the cell membrane, allowing the third to translocate across and induce inflammation and apoptosis (Lin et al., 2011). However, numerous studies have shown that these molecules can modulate the inflammatory response even when administered without the bacteria themselves, demonstrating that these molecules have an independent role in inflammation uncoupled from their role in aiding bacterial adherence or invasion. Flagellin, the protein that forms the filament of the bacterial flagellum, was one of the first molecules to be shown to have this effect. *Salmonella*-derived flagellin was found to have a dose-dependent pro-inflammatory effect in mice, even causing death at the highest doses, despite being administered without the pathogen itself (Ciacci-Woolwine et al., 1998; Eaves-Pyles et al., 2001). Later, it was demonstrated that *Salmonella*-derived flagellin activated the NLRC4 inflammasome by neuronal apoptosis inhibitory proteins (NAIPs) (Miao et al., 2010; Bouwman et al., 2014). Since then, similar inflammatory responses have been reported to be elicited by flagellin derived from other bacterial strains, such as commensal *E. coli* strains (Rhee et al., 2005) and enterohemorrhagic *E. coli* (Lewis et al., 2016). Presumably, these molecules can elicit a pro-inflammatory response because the immune system has learned to recognize a foreign invader from its cell surface, and thus mounts a defense as soon as the intruder is sensed and before they have time to invade host cells and do damage. However, despite being a highly conserved protein across the prokaryotic kingdom, not all bacterial-derived flagellin proteins elicit this same effect. Flagellin derived from *Roseburia intestinalis*, unlike that from pathogenic bacteria, actually has a protective effect on colon epithelial cells *in vitro* and protects from colitis *in vivo* by upregulating the lncRNA HIF1A-AS2, which in turn suppresses the pro-inflammatory response (Quan et al., 2018; Wu et al., 2020).

Other bacterial–cell surface molecules have also been found to have potent anti-inflammatory effects. In mice, strains of both *Bifidobacterium breve* and *Bacillus subtilis* that produce a surface-associated exopolysaccharide are able to evade the host’s immune system, suppress inflammation and thus help protect against *Citrobacter rodentium*-induced enteric insults *via* TLR4 signaling (Fanning et al., 2012; Jones et al., 2014). *Faecalibacterium prausnitzii*, a bacterial species typically underrepresented in patients with inflammatory diseases (Table 1), also has anti-inflammatory effects in experimental models. In both *in vitro* culture and a preclinical mouse model of colitis, two different *F. prausnitzii* strains were able to attenuate the pro-inflammatory response and protect against disease progression (Rossi et al., 2015). Interestingly, the *F. prausnitzii* strain HTF-F, which produces an extracellular polymeric matrix (EPM), was the most successful of the two strains at protecting against inflammation, and treatment with the EPM on its own was sufficient to elicit a protective, anti-inflammatory response (Rossi et al., 2015).

Similarly, *Bifidobacterium longum* 3564, formerly known as *Bifidobacterium infantis*, can attenuate the pro-inflammatory response elicited by pathogenic bacteria both *in vitro* and *in vivo*, not by interfering with their ability to bind and invade intestinal cells (O’Hara et al., 2006), but rather by directly interfering with chemokine secretion and the subsequent pro-inflammatory response to infection (O’Mahony et al., 2008; Sibartie et al., 2009; Scully et al., 2013). This strain was also found to be able to suppress pro-inflammatory chemokines released upon exposure to *Salmonella typhimurium, Clostridium difficile*, or *Mycobacterium paratuberculosis in vitro* (Sibartie et al., 2009), as well as protect against ovalbumin respiratory allergy–induced inflammation in mice (Lyons et al., 2010). It was subsequently demonstrated that this strain differs from other *Bifidobacterium longum* strains in the expression of a unique cell surface exopolysaccharide (sEPS) (Altmann et al., 2016). Not only was this sEPS shown to be crucial in the protective and anti-inflammatory properties of *B. longum* 35624, but removing the gene encoding for this sEPS actually caused the strain to induce a pro-inflammatory response in the lungs when delivered intranasally to mouse models of inflammatory diseases, although polysaccharide-negative strains were not sufficient to induce chronic inflammation and colitis in healthy mice (Schiavi et al., 2016). Since its discovery, studies have shown that treatment with this sEPS alone was sufficient to alleviate inflammation and protect against symptoms in preclinical mouse models of respiratory allergies (Schiavi et al., 2018), osteoporosis (Wallimann et al., 2021) and in mice infected with a lethal influenza virus (Groeger et al., 2020). These anti-inflammatory patterns were also replicated in both healthy volunteers and multiple patient groups fed with *B. longum* 35624 (Konieczna et al., 2012; Groeger et al., 2013; Zaharuddin et al., 2019), though clinical trials involving treatment with sEPS itself are still lacking.

Although *F. prausnitzii* and *Bifidobacterium* spp. are usually underrepresented in pathological contexts, and thus are almost always considered to be “healthy” bacteria, some bacterial species can have opposing effects on inflammation depending on their extracellular chemistry. For example, polysaccharide A (PSA) produced by *B. fragilis* is sufficient to reverse the CD4^+^ T cell deficiency and abnormal lymphoid organogenesis found in germ-free mice, indicating an essential role for an extracellular molecule produced by commensal bacteria in the gut in immune system development (Mazmanian et al., 2005). PSA is also sufficient to suppress interleukin-17 and protect from experimental colitis in a mouse model infected with *Helicobacter hepaticus*, again by acting on CD4^+^ T cell populations (Mazmanian et al., 2008). A follow-up study further elucidated that *B. fragilis*-produced PSA regulates intestinal inflammation by mediating the conversion of CD4^+^ T cells into Foxp3^+^ T cells, which release anti-inflammatory cytokines and protect the intestinal mucosa. Finally, *B. fragilis* can both prevent and cure colitis in mouse models by releasing PSA in outer membrane vesicles, stimulating plasmacytoid dendritic cells to act in concert with CD4^+^ T cells and conferring immunoprotection (Round and Mazmanian, 2010; Shen et al., 2012; Dasgupta et al., 2014). Since these discoveries, PSA has also been shown to confer a protective effect on mouse models of demyelinating disease (Ochoa-Repáraz et al., 2010), viral encephalitis (Ramakrishna et al., 2019), and adverse drug reactions to voriconazole (Wang et al., 2021). As of this time, no clinical studies have been conducted in humans to address whether or not PSA-positive *B. fragilis* could have a protective effect. One possible reason is that, as in the case of *B. fragilis*, the same species of bacteria can have opposing effects on inflammation and disease progression, and thus a great amount of caution must be taken in designing such studies. Apart from PSA, some *B. fragilis* strains can also secrete a tumorigenic toxin referred to as Bacteroides fragilis toxin (BFT), and strains producing this toxin can cause colitis and tumorigenesis in mice (Wu et al., 2009). In humans, PSA-positive *B. fragilis* were significantly reduced in isolates from human patients with IBD, and *B. fragilis* subpopulations expressing BFT were less likely to also be PSA-positive (Blandford et al., 2019), though no human studies have been conducted to address the causality of either BFT or PSA on disease state. Differences in administration between studies are also a variable that should not be underestimated. For example, one study in a mouse model of type 1 diabetes showed that, while oral administration of heat-killed *B. fragilis* had a protective, anti-inflammatory effect, intravenous injection, or oral administration under enhanced gut permeability conditions, actually aggravated the symptoms (Sofi et al., 2019). Notably, *B. fragilis* lacking in PSA had no effect on disease progression, no matter the method by which it was administered (Sofi et al., 2019). Although this further strengthens the evidence for PSA as the driver for *Bacteroides fragilis*-mediated changes, it also suggests that PSA can elicit unforeseen consequences if found in the wrong anatomical compartment. Given the dual role that *B. fragilis* can have on inflammation, and given the beneficial effects of PSA in multiple preclinical mouse models, a perhaps safer therapeutic strategy could be to investigate whether administration of PSA alone is safe, well-tolerated, and anti-inflammatory in humans as well.

Several studies in genetically susceptible germ-free mouse models have elucidated a causal link between *E. faecalis* and IBD by demonstrating that the colonization of the alimentary tract with this single species led to chronic inflammation and disease (Balish and Warner, 2002). A follow-up study in the same model demonstrated that this effect was dependent on the matrix metalloprotease gelatinase, which contributes to chronic inflammation by compromising epithelial barrier integrity (Steck et al., 2011). Surprisingly, however, in a mouse model of IBD, heat-killed *E. faecalis* was found to alleviate inflammation and partially mitigate disease progression (Choi et al., 2019). While the authors of the study demonstrated a dose-dependent decrease in the expression of pro-inflammatory cytokines upon administration of heat-killed *E. faecalis*, they did not address what specific compound was responsible for this anti-inflammatory effect. Despite this lack of mechanistic knowledge, this study strongly suggests that *E. faecalis* can also produce a different compound that has therapeutic potential and is yet another example of how further investigation into the anti-inflammatory effects of bacteria-derived molecules could uncover novel therapeutic strategies, especially where probiotic intervention would be inappropriate.

*Ruminococcus gnavus* is another species often associated with inflammatory bowel diseases, presumably due to its ability to degrade human secretory mucin and thus alter its intestinal microenvironment to make it more favorable to a pro-inflammatory bacterial signature (Table 1 and Png et al., 2010). This bacterial species also produce a glucorhamnan polysaccharide which, when administered alone, can itself induce a potent inflammatory response *in vitro via* TLR4 signaling (Henke et al., 2019; Haynie et al., 2021). On the other hand, a study comparing *R. gnavus* isolates from patients with IBD found that some strains possessed a thick polysaccharide capsule. Bacterial strains lacking this protective capsule induced a strong pro-inflammatory response both *in vitro* and in mice, while those possessing it did not, although whether the polysaccharide capsule impacted the bacteria’s mucolytic abilities was not investigated (Henke et al., 2021). These studies further highlight the need for biological mechanistic insight into the relationship between microbes and disease, especially when trying to interpret correlative clinical studies, or isolating bacterial strains to use in therapeutic scenarios.

## Bacterial metabolites produced in the gut can influence inflammation elsewhere

In addition to expressing molecules on their cell surface, bacteria in the intestine also produce metabolites, which can have implications that are far-reaching throughout the human body. Arguably, the most studied of these metabolites are short chain fatty acids (SCFAs), such as acetate, propionate, and butyrate, which are produced upon bacterial fermentation of dietary fiber and which largely influence inflammation *via* binding to G-protein-coupled receptors (Nogal et al., 2021). The almost ubiquitous trend of reduced *Bifidobacterium* spp. found in the GM of patients with chronic inflammation (Table 1) is often also correlated with a decrease in SCFAs, and which have had some success in alleviating symptoms of IBD (Kanauchi et al., 2002). In mouse models of colitis, asthma, and arthritis, SCFAs attenuated chronic inflammation by directly binding the G-protein-coupled receptor 43 (GPR43) and provoking a strong anti-inflammatory response, indicating that bacteria-derived metabolites can also alleviate inflammation far from the organ in which they live (Maslowski et al., 2009). In mice, *Bifidobacterium lactis* probiotic Probio-M8 reduced Aβ-plaque burden and improved cognition in a mouse model of Alzheimer’s disease (Cao et al., 2021). In humans, patients suffering from ulcerative colitis, psoriasis, or chronic fatigue syndrome all had reduced inflammatory markers when fed *B. infantis* 35624 compared with placebo-fed controls, indicating that these far-reaching anti-inflammatory effects are also reproducible in human subjects (Groeger et al., 2013).

*Clostridium butyricum*, most studied for its production of the SCFA butyrate, has also successfully protected both mouse models and human patients suffering from IBS-induced inflammation, as well as a mouse model of intestinal cancer, when administered as a probiotic (Sun et al., 2018; Zhao et al., 2019; Chen et al., 2020a). Beyond the gut, probiotic use of *C. butyricum* was also found to be protective against inflammation in mouse models of acute pancreatitis (Pan et al., 2019a), atherosclerosis (Chen et al., 2020b), metabolic disorders (Stoeva et al., 2021), and multiple sclerosis (Chen et al., 2019). While probiotic use of *C. butyricum* is believed to act on inflammation at many different levels, from “correcting” dysbiosis in the intestine to reducing intestinal leakage and beyond (Stoeva et al., 2021), the administration of sodium butyrate alone has also demonstrated an anti-inflammatory effect conferred directly by this bacterial metabolite. In a mouse model of Crohn’s disease, sodium butyrate feeding before TNBS-induced colitis significantly protected mice from inflammation and intestinal barrier dysfunction by binding G-protein-coupled Receptor 109 A (GPR109A), inhibiting histone deacetylases and suppressing pro-inflammatory pathways (Chen et al., 2018; Dou et al., 2020). Furthermore, butyrate produced by bacteria in the gut can enter the bloodstream, travel to multiple organs, and even cross the blood–brain barrier (Liu et al., 2020). Sodium butyrate feeding has thus also been shown to have an anti-inflammatory effect and protect against symptoms in mouse models of depression (Qiu et al., 2020), pancreatitis (Pan et al., 2019b), kidney disease (Felizardo et al., 2019), and obesity (Hong et al., 2016). Interestingly, sodium butyrate supplementation had an opposite effect when administered during gestation, with rats born to butyrate-fed mothers presenting with insulin resistance and increased skeletal fat accumulation (Huang et al., 2017), suggesting that SCFA metabolism during pregnancy can have very different consequences on health and disease outcome.

*Faecalibacterium prausnitzii* is another butyrate-producing commensal species found in abundance in the healthy human intestine, with strain-specific differences in butyrate production that correlate with its anti-inflammatory strength (Martín et al., 2017). Studies have found that the treatment of preclinical rodent models of colitis with both the bacteria itself and its culture supernatant has an anti-inflammatory effect, suggesting that secreted molecules, likely butyrate, are responsible for conferring protection against colitis (Qiu et al., 2013; Martín et al., 2014). Similarly, both living and dead preparations of *Faecalibacterium prausnitzii* could alleviate inflammation in a mouse model of asthma, also by directly modulating SCFA production (Hu et al., 2021). *F. prausnitzii* has also been used successfully as an anti-inflammatory prophylactic in a mouse model of pelvic radiation disease (Lapiere et al., 2020) and in a rat model of depression and anxiety (Hao et al., 2019). These studies, together with an almost universal signature of decreased *F. prausnitzii* populations in the GM of patients with inflammatory diseases, have made it an excellent candidate for future human studies and its production as a probiotic, though results in clinical trials are still lacking.

Other SCFAs have also been used, either singularly or together, in mouse models of inflammation beyond the gut. In a preclinical model of non-alcoholic steatohepatitis sodium acetate, sodium butyrate, and sodium propionate were all individually found to protect against inflammation and disease progression, although sodium acetate was the most successful of the three (Deng et al., 2020). Another study in mice fed with a high-fat diet found that the combinatorial effect of acetate and propionate was more effective than butyrate as a suppressor of inflammation, increased body weight, and diabetes (Mandaliya et al., 2021). Methyl acetate, on the other hand, was able to suppress inflammatory cell infiltration in the central nervous system, thereby protecting the spinal cord from demyelination and improving the health of a mouse model of multiple sclerosis (Xie et al., 2021), while ethyl acetate alleviated inflammation and rheumatoid arthritis in rats (Ikram et al., 2021). Propionate has also successfully been used to suppress inflammation and improve disease outcomes in mouse models of colitis (Bajic et al., 2020), atherosclerosis (Haghikia et al., 2022), and hypertensive cardiovascular damage (Bartolomaeus et al., 2019). Taken together, these studies suggest a system-wide anti-inflammatory effect of bacterially derived SCFAs, providing exciting new possibilities for therapy.

Interestingly, the anti-inflammatory properties of SCFA production have not always been associated with a beneficial outcome. One study employing both metagenomic and metabolomic profiling demonstrated elevated levels of both SCFAs and SCFA-producing bacteria in patients with non-alcoholic fatty liver disease and hepatocellular carcinoma (Behary et al., 2021). Furthermore, bacterial extracts from patients elicited an immunosuppressive response in peripheral blood mononuclear cell preparations, suggesting that, by dampening the immune system, GM-derived SCFAs can create a permissive landscape for cancerous cells to prosper by evading immune checkpoints (Behary et al., 2021). Studies like these highlight the importance of comprehending both the biological context and the mechanisms by which the GM can influence disease, to predict potential unintended consequences of modulating the GM in response to pathology.

In addition to dietary fiber, bacteria in the gut also participate in the metabolism of the amino acid tryptophan, producing metabolites such as indole, skatole, and tryptamine derivatives (Gasaly et al., 2021). These metabolites also have been found to regulate the gut microbial community, and intestinal immunity, and can have a systemic-wide effect on inflammation *via* binding to the xenobiotic receptor AhR (Zelante et al., 2013; Gasaly et al., 2021). In mice, *Lactobacillus*-derived tryptophan metabolites protect the intestinal mucosa and suppress inflammation *via* IL-22 (Zelante et al., 2013). *In vitro*, the *Bifidobacterium*-derived tryptophan metabolite indole-3-lactic acid alone was found to be able to suppress TNF-α and IL-8 in chemically stressed gut epithelial cells (Ehrlich et al., 2020). Beyond the gut, tryptophan metabolites have been found to suppress inflammation *via* AHR signaling in the central nervous system (Rothhammer et al., 2016) and have been found to have beneficial effects on diabetes and metabolic syndromes by regulating the microRNA *miR-181* family (Galligan, 2018; Virtue et al., 2019). In humans, tryptophan metabolites in the serum are negatively correlated with disease activity in patients with IBD (Nikolaus et al., 2017) and with waist-to-hip ratio and systemic inflammation in people infected with HIV (Gelpi et al., 2020). However, a diet high in dietary fiber has been shown to have a beneficial effect on children with obesity and people with IBD (Zhang et al., 2015; Fritsch et al., 2021), and while this diet also increased tryptophan metabolites (among many other things), clinical trials investigating the effect of tryptophan catabolism specifically on chronic inflammation are still lacking.

## Modulation of the gut microbiota in human trials has led to mixed results

Fecal microbiota transplantation (FMT) is a clinical practice by which fecal samples from healthy donors are transplanted into patients with severe dysbiosis. While this method is widely used to treat severely ill patients with *C. difficile* infections, the emerging importance of GM health in other pathological contexts has led to clinical trials of FMT in many different inflammatory diseases (Table 3). However, given the lack of deep -omics-driven characterization of the GM in clinical settings, as well as the gaps in mechanistic knowledge that still exist in our understanding of how the GM modulates inflammation, it is understandable that therapeutic intervention *via* FMT has not always resulted in clear-cut success (Table 3). In the case of ulcerative colitis, FMT generally has an approximately 30% success rate, and some success has also been noted for patients with cancer and cirrhosis (Table 3). However, despite the fact that FMT can protect from intestinal inflammation, insulin resistance, and weight gain in a preclinical mouse model of diabetes and diabetic kidney disease, clinical trials of FMT to treat obesity and insulin resistance have not been successful (Bastos et al., 2022 and Table 3).

**TABLE 3:** Overview of different FMT clinical trials and their outcome.

Patient no.	Administration	Disease	Clinical outcome	References
73 (adults)	Enema	UC	12/38 achieved remission within 8 weeks. 3/38 had serious adverse events.	Costello et al., 2019
85 (adults)	Enema	UC	11/41 remission, 2/41 serious adverse events.	Paramsothy et al., 2017
70 (adults)	Enema	UC	24% achieved remission after 7 weeks	Moayyedi et al., 2015
50 (adults)	Nasoduodenal tube	UC	No significant difference in remission	Rossen et al., 2015
41 (adults)	Colonoscopy	UC	No remission after 8 weeks	Nishida et al., 2017
2 (children)	Colonoscopy	UC	1 clinical remission, 1 clinical worsening.	Quagliariello et al., 2020
10 (children)	Enema	UC	78% had clinical response, 33% achieved remission.	Kunde et al., 2013
17 (adults)	Colonoscopy	CD (in remission)	FMT did not prevent relapse.	Sokol et al., 2020
165 (adults)	Gastroscope	IBS	Dose-dependent improvement.	El-Salhy et al., 2020
20 (adults)	Enema	AUD	90% patients decreased alcohol cravings.	Bajaj et al., 2021
18 (children)	Oral and enema	ASD	Significant behavioral and gastrointestinal improvements.	Kang et al., 2017
16 (adults)	Endoscope	PD-1-refractory melanoma	6/15 with clinical benefit.	Davar et al., 2021
24 (adults)	Oral	Obesity + IR	No significant outcome	Yu et al., 2020
87 (adolescents)	Oral	Obesity	No effect	Leong et al., 2020
22 (adults)	Oral	Obesity	No significant difference	Allegretti et al., 2020
20 (adults)	Nasoduodenal tube	Recent-onset T1D	Decline in insulin production was halted at 12 months	de Groot et al., 2021
21 (adults)	Nasoduodenal tube	NAFLD	No effect on liver or IR, but small amelioration of intestinal permeability	Craven et al., 2020
20 (adult men)	Enema	Recurrent HE	Improved cognition, no recurring HE	Bajaj et al., 2017
10 (adults)	Colonoscopy	PSC	No adverse effects	Allegretti et al., 2019
116 (adults)	Oral/Colonoscopy	RCDI	Both >95% efficient and treating RCDI	Kao et al., 2017

AUD, alcohol use disorder; ASD, autism spectrum disorder; ASCD, Crohn’s disease; FMT, fecal microbiota transplant; HE, hepatic encephalopathy; IBS, irritable bowel syndrome; NAFLD, non-alcoholic fatty liver disease; PSC, primary sclerosing cholangitis; RCDI, recurrent clostridium difficile infection; T1D, type-1 diabetes; UC, ulcerative colitis. Unpublished or still ongoing FMT clinical trials can be overviewed at ClinicalTrials.gov.

**FIGURE 1:**
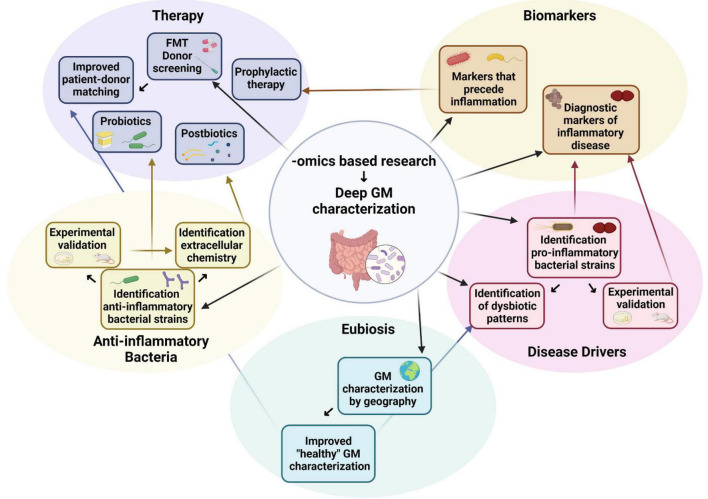
How omics-based research can inform and improve study design and therapeutic strategies. Created with BioRender.com.

In most cases, the side effects of FMT consist of mild to moderate forms of gastrointestinal discomfort. Unfortunately, some of the other risks associated with FMT are, while uncommon, still serious enough to be noteworthy. In the case of ulcerative colitis, some patients receiving FMT have experienced worsening colitis, colectomy, or pneumonia (Paramsothy et al., 2017; Costello et al., 2019; Quagliariello et al., 2020), and one patient receiving FMT for chronic diarrhea developed adhesion ileus (Harsch and Konturek, 2019). Perplexingly, despite the lack of clinical evidence of an effect of FMT on obesity (Table 3), one case study reported a *C. difficile*-infected patient experiencing rapid and inexplicable weight gain after receiving FMT from her own overweight daughter (Alang and Kelly, 2015). In one tragic case, failure to adequately screen the donor material for drug-resistant pathogens led to bacteremia and the death of one recipient of FMT (DeFilipp et al., 2019). While the most severe of these adverse effects can be overcome by a more rigorous screening of donor samples for infectious pathogens, it is still clear that there are additional, currently unknown variables at play when patients undergo FMT. These unknown variables, combined with a broad spectrum of administration methods, dosages, donor–patient matching criteria, and evaluation of transplant success have all likely contributed to the uncertain outcome of FMT in treating inflammatory diseases. With the recent increase in interest in FMT as a therapeutic option for multiple syndromes, it is clear that a certain amount of standardization in sample screening, storage, and administration is necessary for a more robust clinical outcome (Cammarota et al., 2019). However, it is also apparent that more profound knowledge of the biological mechanisms by which the GM modulate inflammation, as well as a deeper -omics-based characterization of both patient and donor fecal samples, could help bring us closer to clinical success (Figure 1).

## Probiotic and metabolite intervention in patients with inflammatory diseases

While FMT has had some success in treating patients with inflammatory diseases, the complexity of the GM has also led to a number of confounding variables that can mask a successful outcome and that have been difficult to identify. With new advances in mechanistic insight, some clinical studies have been able to investigate probiotic intervention in inflammatory diseases, which can be more cost-effective, less invasive, and simpler to evaluate for safety and side effects. In the gut, probiotics were found to reduce disease severity and inflammatory markers in patients with IBS (Xu et al., 2021), as well as suppress inflammation in the intestine of adults and children with cystic fibrosis (Coffey et al., 2020) and in HIV-infected patients receiving antiretroviral medication (d’Ettorre et al., 2015). Beyond the gut, probiotics have also been found to alleviate inflammation and ameliorate symptoms of diseases of various etiology. In the context of metabolic syndromes, such as obesity, non-alcoholic fatty liver disease, type-2 diabetes, gestational diabetes, chronic kidney disease, and insulin resistance, numerous clinical studies have found a beneficial effect of probiotic intervention on inflammatory biomarkers, body mass index (BMI), intrahepatic fat, and insulin sensitivity (Sáez-Lara et al., 2016; Hajifaraji et al., 2018; Ahn et al., 2019; Vlachou et al., 2020; Shirvani-Rad et al., 2021; Zheng et al., 2021). Taken together, these studies suggest that the failures of FMT intervention in metabolic syndromes were due more to issues in methodology, rather than the result of the GM not being a driver of metabolic diseases. On the other hand, studies investigating probiotics as a treatment for neuropsychiatric disorders have been less conclusive. For example, while a meta-analysis of five clinical trials found that probiotic intervention decreased inflammatory biomarkers and improved cognitive impairment in patients with Alzheimer’s disease and mild cognitive impairment (Den et al., 2020), a different meta-analysis concluded that there is insufficient evidence to support the use of probiotics as a co-treatment for dementia (Krüger et al., 2021). Furthermore, probiotics had little to no impact in a clinical trial of people with mild to moderate depression, although a lack of an effect on the GM after probiotic use suggests that this may have been due to an insufficient dosage (Chahwan et al., 2019). Similarly, one meta-analysis suggested that *Lactobacillus*-laced probiotics were an effective adjunct treatment for patients with chronic periodontal disease (Matsubara et al., 2016), while another found the statistical evidence to this effect to be unreliable (Yanine et al., 2013).

So far, the biological evidence has uncovered specific bacterial species as of particular importance in suppressing inflammation in the intestine, such as lactic acid–producing bacteria and Bifidobacteria (Saez-Lara et al., 2015). Therefore, many clinical studies have focused on treatment with one or two bacterial strains instead of employing highly variable multi-strain probiotics. For example, *Lactobacillus casei* alleviated symptoms of acute diarrhea in affected children (Lai et al., 2019), while *Bifidobacterium longum* NCC3001 had no effect on IBS symptoms but did alleviate depression in patients with IBS (Pinto-Sanchez et al., 2017). In the brain, *Lactobacillus plantarum* PS128 seems to have a synergistic effect with oxytocin to help social cognition responses in patients with autism (Kong et al., 2021), while *Bifidobacterium breve* had some positive effects on memory in elderly patients with mild cognitive impairment (Xiao et al., 2020). Furthermore, probiotics in general and a combination *Bifidobacterium bifidum* BGN4 and *Bifidobacterium longum* BORI in particular reduced chronic low-grade inflammation and promoted mental flexibility in healthy elderly adults (Custodero et al., 2018; Kim et al., 2021), though no effect on inflammation or mood was found when depressed patients were treated with *Lactobacillus helveticus* and *B. longum* (Romijn et al., 2017). *Bifidobacterium lactis* was also found to help as an adjuvant therapy in reducing inflammation in patients with asthma (Liu et al., 2021). While this approach addresses issues of high variability between multi-strain probiotics, in some cases, treatment with a single strain is not sufficient to elicit the desired robust clinical outcome. For example, while one small study employing *Bifidobacterium*-fermented milk showed promising results in patients with ulcerative colitis (Ishikawa et al., 2003) and despite the strong preclinical data described above, a meta-analysis of five studies in patients with IBS reported that *B. longum* 35624 was only efficient in alleviating symptoms when administered with other probiotic bacteria (Yuan et al., 2017).

Despite being less expensive and invasive, clinical studies with probiotic therapy have suffered from many of the same issues as those with FMT, including small patient cohorts, high variability in dosage, and inconsistent monitoring of the GM response to probiotic use. Furthermore, while probiotic treatment is associated with fewer recorded adverse effects than FMT, and despite having been found to have had some success in modulating inflammation in patients with gastric cancer (Yang et al., 2022), probiotic intervention has been linked to rare cases of sepsis in patients with cancer, thus requiring more caution in clinical trials involving the immunocompromised (Redman et al., 2014).

In addition to probiotics, clinical trials have also been conducted with postbiotics, i.e., bacteria-derived metabolites, in patients with inflammatory diseases. However, despite very promising data in rodent models, supplementation with individual SCFAs has had less success in human clinical trials. While consumption of sodium butyrate has been determined to be safe for humans, multiple clinical trials in patients with IBD have resulted in little to no amelioration of symptoms, nor was it effective as a therapeutic strategy in type 1 diabetes (Steinhart et al., 1996; de Groot et al., 2020; Facchin et al., 2020; Jamka et al., 2021). However, these clinical trials differed from studies in animal models in a few key ways. First of all, sodium butyrate was administered to patients with IBD by enema, whereas the previous studies in mice were conducted by butyrate feeding, and another mouse study demonstrated diametrically opposing effects on inflammation by butyrate enema depending on the dosage used (Liu et al., 2020). While the clinical trial conducted with patients with diabetes also administered butyrate orally, it is important to note that all of the previously mentioned preclinical studies demonstrated a protective effect of butyrate when administered before the inflammatory insult occurred. More studies are needed to determine whether, in humans too, butyrate could be used as an effective prophylactic against inflammatory diseases.

## How -omics-based characterization and mechanistic insight could shape the future of clinical trials

Since the diversity, complexity, and responses of the gut microbiota to disease states became apparent, much controversy has revolved around whether these microbial communities were markers of disease, or whether they were one of, if not the main drivers of pathology. In recent years, numerous studies have uncovered a causal link between the GM and inflammatory diseases, with our knowledge of the biological mechanisms they employ to do this having come forward by leaps and bounds. Despite these advances, there still exist some significant gaps, both in our understanding of how these bacteria influence disease and in our ability to bridge the gap between the microbiology laboratory and the clinical world, with human trials ending in far less conclusive victories than many of their *in vitro* or preclinical rodent model counterparts. While it is well known that these models are oversimplified and thus can never fully replicate a human patient, some of these clinical disappointments might, at least in part, be due to unknown and unforeseen confounding factors. In a clinical setting, the biological mechanism *via* which these bacteria might influence inflammation is often overlooked. While this point of view is understandable in a diagnostic context, where the presence of a certain bacteria can be seen as a pathological red flag regardless of the “why,” it can also limit our ability to develop novel therapeutic strategies.

One of the biggest issues standing in the way of bridging the gap between models and humans is the relatively superficial information that is gleaned from microbiotic profiling of human patients. While laboratory studies on individual bacterial species can have the luxury of a deep characterization of the bacteria in question, down to the peculiarities of the strain and its extracellular chemistry, GM-profiling of human stool samples usually involves identifying the bacteria found at the family or at most the genus level. These methods can give a general overview of the state of the GM in human subjects but is not sufficient to identify specific bacterial markers or drivers of inflammatory diseases, especially in light of the discovery that different strains of the same species can have diametrically opposite effects on inflammation. Given the large heterogeneity uncovered by studies into the biological mechanisms of bacteria-induced inflammation, it is therefore understandable that association studies in humans lacking strain-level information can sometimes be difficult to interpret or reproduce. On the other hand, -omics-based methodologies currently available to characterize the microbiome, exposome, and immunome of patients have improved dramatically in recent years, bringing us ever closer to novel personalized medicine interventional strategies (Figure 1 and Putignani et al., 2019). It must also be noted that, though bacteria have historically been the largest focus of studies in microbiome research, the healthy GM is also composed of viruses, fungi, and archaea that have their own regulatory role in inflammation (Norman et al., 2014). While including these modulators of chronic inflammation was unfortunately beyond the scope of this review, a deeper characterization of the microbiome in this precision medicine model would also ideally not exclude these important players, to better bridge this gap between the lab and the clinic.

This in-depth characterization of the biological mechanisms by which bacteria modulate inflammation has brought to light another significant issue in current clinical trial design. In the case of FMT, donors are selected based on age, gender, and overall metabolic health, and samples are screened for pathogens, but usually little is known about the microbial composition of their GM. While this may be sufficient characterization in the treatment of *C. difficile* infection, it is likely not enough to adequately choose the most appropriate donor for patients with inflammatory diseases, likely contributing to the uncertain outcomes described previously. Similarly, there is also the often-overlooked variability in the production of probiotics, especially those which are anaerobic. For example, *F. prausnitzii*, which has gained much interest in recent years as a next-generation probiotic, is also very sensitive to oxygen, which can make the purification of enough live bacteria to make a difference when ingested very tricky (Kim et al., 2020). Variability between production methods can often lead to not only differences in viability of the bacteria but also in effector molecule production, which can be destroyed by industrial methods and are virtually never taken into consideration as quality control markers for probiotic production (Duboux et al., 2021). Given the anti-inflammatory importance of specific bacteria-derived molecules that has come to light in recent years, and which has been discussed previously in this review, a lack of standard protocols in ensuring the integrity of these molecules during probiotic production could well mask beneficial effects on inflammatory diseases conferred by these microbes. Furthermore, in the case of both FMT and probiotic use, standard protocols are not only needed in how the therapeutic agent is treated but also in the confirmation that the therapy had a measurable effect on the GM of the recipient. If the FMT did not graft, or if the probiotic dosage used was too low to ensure the survival and colonization of the bacteria in the gut, then the therapy does not have any chance of having a protective effect. In the case of probiotics, they are sometimes administered in clinical trials together with various non-digestible fiber sources, known as prebiotics, which adds yet another layer of variability between different clinical trials that can make outcomes difficult to interpret or reproduce.

Biological life is inherently variable, which is why those who study it are obligated to adapt their protocols to fit the scenario and employ different statistical methods to ensure that differences measured are significant. However, when high variability is not compensated for by large sample sizes, many potentially significant results can be masked by it. Additionally, the vast methodological variability between different clinical trials has undoubtedly contributed to the lack of reproducibility of some clinical outcomes. While a certain amount of flexibility must exist in clinical trial design to adjust the treatment to the circumstances, a certain amount of standardization could help to reduce uncertainty due to unnecessary variability in study design.

Another source of variability that is not often discussed is the impressive difference in GM composition between individuals. Though it is well known that diet has a strong effect on the gut microbiota, one could argue that a healthy microbial community is associated with a healthy diet, and thus that diet-induced changes to this ideal GM could be considered unhealthy, or dysbiotic. However, geographical location is another important factor that is often overlooked. One study demonstrated that the microbiota of Colombian adults possessed a different microbial signature than that of Americans, Europeans, and Asians, as well as a different set of microbes altered in obese Columbian individuals (Escobar et al., 2014). Consistently, a study on fecal samples from healthy United States and Spanish participants found a significant difference in GM diversity overall and of specific bacterial genera in particular between the two nationalities (Allali et al., 2015). Even within the same country, GM composition was found to be the number one predictor of whether a person came from Northern or Southern China, with geography having a far larger influence on the GM than ethnicity (Lu et al., 2021; Wang et al., 2022). While the diets of different populations in different parts of the world undoubtedly contribute to these differences, these studies still beg the question: what is a healthy GM? Could a “healthy” GM characterization conducted in the United States really be used as a model for a “healthy” European, Chinese, or Brazilian person? When we identify dysbiosis in patients with inflammatory diseases, what are we comparing them too? Given recent evidence, it is far more likely that there are many different compositions of the GM that have evolved over time and that are “healthy,” based on the climate, diet, and genetics the person finds themselves with. Greater consideration of these differences between human populations must be taken, as well as an expansion of available geographically specific data on human GM composition (Figure 1).

As with the majority of human ailments, there is no “one size fits all” answer to the question of how we can alleviate inflammation *via* the GM, with each method having its up- and downsides. Methods such as FMT and multispecies probiotics have the benefit of taking into account the larger biological picture, since many bacteria need to be balanced and act in concert with others in order to elicit a protective effect. However, this strength can also be considered its greatest weakness, since a lack of standard operating procedure and superficial characterization of the donor/probiotic material also introduces a variability between patients that is not always possible to overcome statistically. On the other hand, treatment with single probiotics or metabolites can help overcome these statistical challenges but not always be effective enough to act on their own. Instead of attempting to find a solution to all of these problems at once, one should instead use the mechanistic knowledge gained to best inform their clinical study design. For example, upon the discovery of the importance of *B. longum* 3564-derived sEPS in inflammation, any clinical trial conducted with this strain should take the integrity of its extracellular chemistry during probiotic production as a fundamental quality control parameter. With the discovery of the dual role of *B. fragilis* in inflammation based on a series of different effector molecules it can produce, a safer and more intriguing clinical trial could be conducted on the efficacy of PSA itself, rather than risk worsening symptoms by attempting to treat patients with an insufficiently well-characterized *B. fragilis* strain. Although designing a clinical trial for every single promising effector molecule is unreasonable, time-consuming, and expensive, the same can be said when FMT- or probiotic-based clinical trials are designed and conducted only to yield inconclusive results. A more well-rounded approach that takes biological mechanism into consideration could help identify key pitfalls and help to design better, more robust clinical studies.

## Discussion

It is clear that, at least in some cases, the GM can modulate inflammation both in the intestine and beyond. However, even if, in some disease contexts, dysbiosis is more a marker than a driver of inflammation, there is still much to be gained by a deeper understanding of microbial markers in inflammatory diseases. For example, when does dysbiosis in the gut occur? Does it precede disease onset, or does it occur after symptoms present themselves? Can we, in short, employ our ever-expanding -omics repertoire to design longitudinal studies, with the aim of identifying early microbial markers of inflammatory diseases? By combining our mechanistic knowledge of microbially modulated inflammation with a deeper -omics-based patient GM characterization, designing more personalized therapeutic or prophylactic interventions becomes ever more within our reach (Figure 1).

## Author contributions

AP and LP wrote the review. Both authors contributed to the article and approved the submitted version.
